# Neurochemical changes in animal models of Parkinson’s disease

**DOI:** 10.1186/1750-1326-8-S1-P7

**Published:** 2013-09-13

**Authors:** Mária Baranyi, Elisaveta Milusheva, Beáta Sperlágh

**Affiliations:** 1 Laboratory of Molecular Pharmacology, Institute of Experimental Medicine, Hungarian Academy of Sciences, Laboratory of Molecular Pharmacology, Institut H-1450 Budapest, PO.Box 67, Hungary; 2 Institute of Physiology, Bulgarian Academy of Sciences, Acad. G. Bonchev Street, BL. 23, 1113 Sofia, Bulgaria

## 

Parkinson’s disease (PD) is a neurodegenerative syndrome associated with selective loss of dopaminergic neurons in the striatum. It is believed that both mitochondrial dysfunction and oxidative stress (OS) play important role in the pathogenesis of PD. Development of an effective causal therapy should be focused on preventing or at least retarding the neurodegenerative process underlying the disease.

In chronic in fusion PD model studied the effect of rotenone on the content, and release of striatal neurotransmitters of rat upon OS by H2O 2 perfusion. The effects of anti-Parkinsonian drugs were examined *in vitro* rotenone PD, OS slices model. The role of P2X7 receptor (P2X7r) formed by 1-methyl-4-phenyl-1,2,3,6- tetrahydropyridine (MPTP) or *in vitro* rotenone treatment using C57/BI6 and P2X7r knockout mice. Neurochemical response was determined using microvolume perfusion method, HPLC separation of dansylated samples. Dopamine o-quinone (DAQ) oxidized metabolite of DA was detected electrochemically at -100 mV potential in reduction working mode.

The dopamine content in the rat striatum is decreased in response to chronic intravenous rotenone infusion. However, surviving dopaminergic neurons take up and release only a slightly lower amount of DA in response to electrical stimulation. Striatal dopaminergic neurons showed increased susceptibility to OS, responding with enhanced release of DA and with formation of toxic metabolite DAQ. The loss of DA and ATP and formation of DAQ induced by OS confirmed the impairment of striatum upon in vitro rotenone. Formation of DAQ at the end effect of OS confirmed the impairment of dopaminergic function by rotenone. However, when applied rotenone with l-DOPA treatment the endogenous DAQ was detected in perfusion samples. However, when applied rotenone with l-DOPA treatment the endogenous DAQ was detected in the micro-volume perfusion samples. Conversely, when rasagiline and trolox were in use these DA-quinones metabolites were not detectable. Rotenone treatment elicited a similar reduction in ATP content in the substantia nigra of both genotypes, whereas reduction of ATP was more pronounced after rotenone treatment in striatal slices of P2X7 deficient mice. Although the level of the endocannabinoid, 2-AG, was elevated by rotenone in the striatum of wild-type mice, an effect that was absent in mice deficient in P2X7 receptors. Genetic deletion of P2X7 receptors did not change depletion of striatal endogenous DA content after *in vivo* MPTP or *in vitro* rotenone treatment. Although the endogenous amino acids content and the level of the endocannabinoid, AEA was elevated by MPTP in the striatum of mice, but 2-AG remained unchanged in mice deficient in P2X7 receptors.

In conclusion, oxidative stress induced, pathological DA release and the formation of toxic metabolites of DA are distinctly modified by the tested antiparkinsonian drugs and P2X7 receptor deficiency does not promote the survival of dopaminergic neurons.

